# Supplementary material for: Deletion of wfs1 Impairs Oligodendrocyte Precursor Cells Dorsal Distribution and Myelination Through the wfs1-hmgcs1 Axis in Zebrafish
Source: Biology (Basel). 2026 Aug 16;15(16):1402. doi: 10.3390/biology15161402 (PMC13510151; doi:10.3390/biology15161402)
Supplement: Supplementary file 1 [file biology-15-01402-s001.zip › File S1-Transcriptome sequencing data and differentially expressed genes (DEGs) in wfs1-- zebrafish at 5 dpf/1.GO/wfs1KOvsWT/UP/wfs1KOvsWT.up_GOdot.pdf]

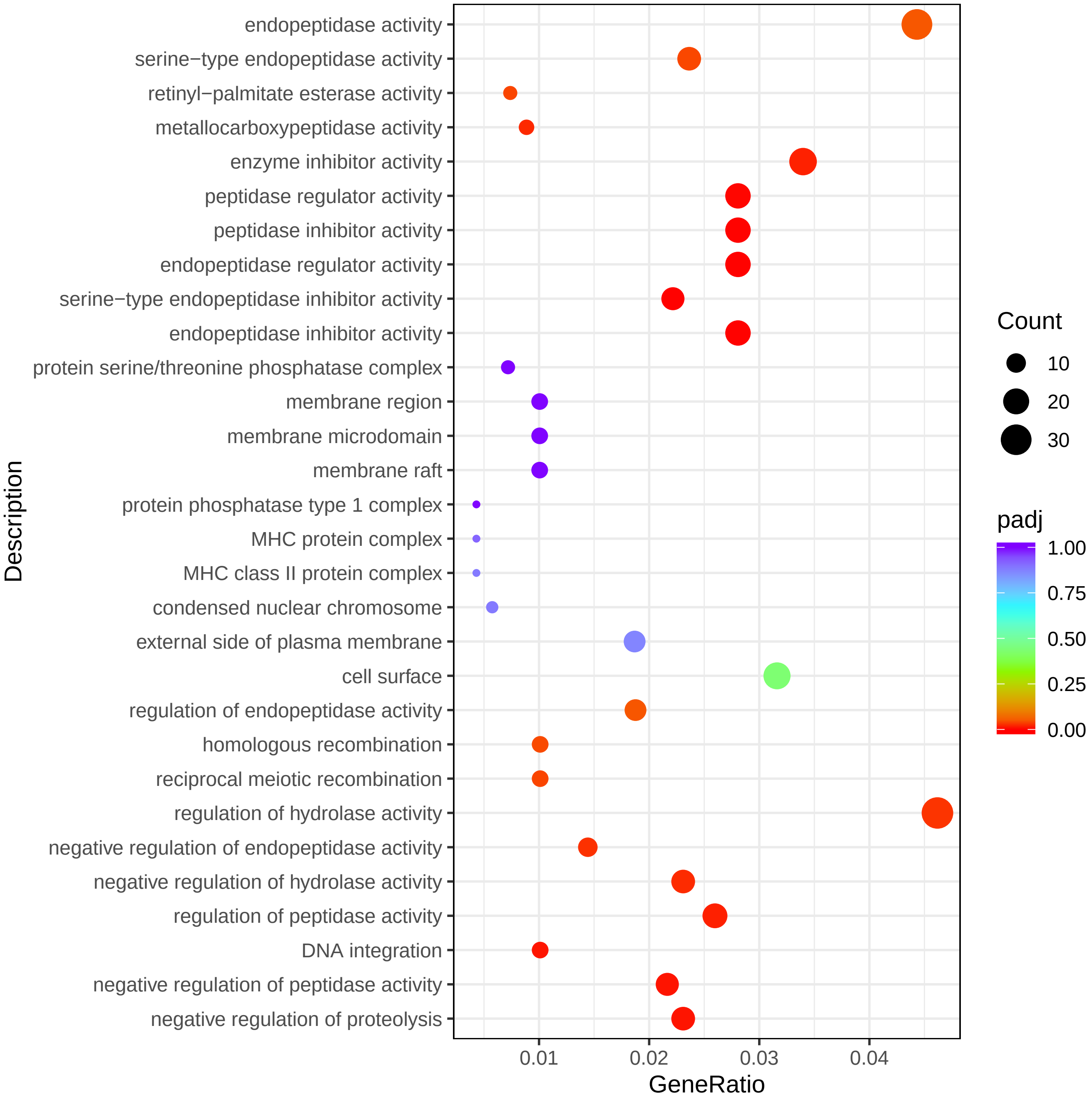
endopeptidase activity
serine−type endopeptidase activity
retinyl−palmitate esterase activity
metallocarboxypeptidase activity
enzyme inhibitor activity
peptidase regulator activity
peptidase inhibitor activity
endopeptidase regulator activity
serine−type endopeptidase inhibitor activity
endopeptidase inhibitor activity
protein serine/threonine phosphatase complex
membrane region
membrane microdomain
membrane raft
protein phosphatase type 1 complex
MHC protein complex
MHC class II protein complex
condensed nuclear chromosome
external side of plasma membrane
cell surface
regulation of endopeptidase activity
homologous recombination
reciprocal meiotic recombination
regulation of hydrolase activity
negative regulation of endopeptidase activity
negative regulation of hydrolase activity
regulation of peptidase activity
DNA integration
negative regulation of peptidase activity
negative regulation of proteolysis
Description
0.01
0.02
0.03
0.04
GeneRatio
Count
10
20
30
padj
1.00
0.75
0.50
0.25
0.00
